# Uremic Leontiasis Ossea in a Pediatric Patient

**DOI:** 10.7759/cureus.23428

**Published:** 2022-03-23

**Authors:** Hisham K Algossy, Fahad Albadr, Abdullah Y Aloraini, Mohammed K Alsayyar, Abdullah H AlHindi

**Affiliations:** 1 Radiology, King Saud Medical City, Riyadh, SAU; 2 Radiology and Medical Imaging/Neuroradiology, King Saud Medical City, King Saud University, Riyadh, SAU; 3 Radiology, Buraidah Central Hospital., Buraidah, SAU

**Keywords:** cranial facial bone thickening, paget's disease, fibrous dysplasia, deformity, visual loss, hearing loss, ossification of the temporal bone, lion like face, hyperparathyroidism, end stage renal disease (esrd)

## Abstract

Uremic leontiasis ossea (ULO) is a rare disease characterized by extensive thickening of the cranium, resulting in a characteristic, lion-like facial appearance. It is considered the most severe osseous complication of renal dystrophy. Although rare, ULO can occur even in young patients, which can be catastrophic, as it can not only lead to life-threatening conditions but also multiple complications that cause severe determent to the quality of life.

## Introduction

End-stage kidney disease (ESKD) is a serious disease with many known associated comorbidities and complications, including secondary hyperparathyroidism, which in severe cases can lead to uremic leontiasis ossea (ULO) [1]. Leontiasis ossea refers to the distinctive lion-like appearance that occurs as a result of the hyperostotic thickening of the cranium, regardless of the cause [2]. In addition to ESKD, leontiasis ossea can occur because of various conditions, including Paget’s disease, acromegaly, and McCune-Albright syndrome [3]. Leontiasis ossea related to ULO or other causes is important not only because of the cosmetic impacts of the disease but also for the more concerning complications, such as extension into the nasal, oral, and even orbital areas, as a result of the progressive thickening of the facial bones. In severe cases, it can even lead to airway obstruction for similar reasons [4]. Knowing that ULO occurs secondary to hyperparathyroidism in patients with ESKD is critical in the diagnosis, as ULO has clinical, histological, and radiological similarities with several fibro-osseous conditions involving the craniofacial region, which can be approached differently [5].

## Case presentation

An eight-year-old-girl patient with ESKD developed enlarged bony structures, including the skull and jaw, and facial disfigurement, along with other features including diffuse bone pain. She had an obvious bony expansion of the maxilla and mandible as well as gingival hyperplasia on examination. The patient also had oral dysphagia, reduced oral control, and reduced mouth opening. Hard submandibular swelling was observed, and the tongue was found to be pushed up because of the hard swelling. Moreover, she had recurrent choking episodes. On admission, laboratory test results confirmed tertiary hyperparathyroidism and were as follows: corrected calcium level, 2.68 mmol/L; phosphorus level, 2.19 mmol/L; alkaline phosphatase level > 1000 U/L; and parathyroid hormone (PTH) level > 201 pmol/L. Phosphorus and PTH were clearly increased, alkaline phosphatase (ALP) was clearly elevated, and corrected calcium was on the upper limit of normal (Table 1).

**Table 1:** Lab values of the patient: corrected calcium, phosphorus, alkaline phosphatase, and parathyroid hormone (PTH) upon admission Normal range is provided.

	Corrected Calcium (mmol/L)	Phosphorus (mmol/L)	Alkaline Phosphatase (U/L)	PTH (pmol/L)
Patient result	2.68	2.19	>1000	>201
Normal value	2.20-2.70	1.45-1.78	156-369	1.18-8.43

**Figure 1:**
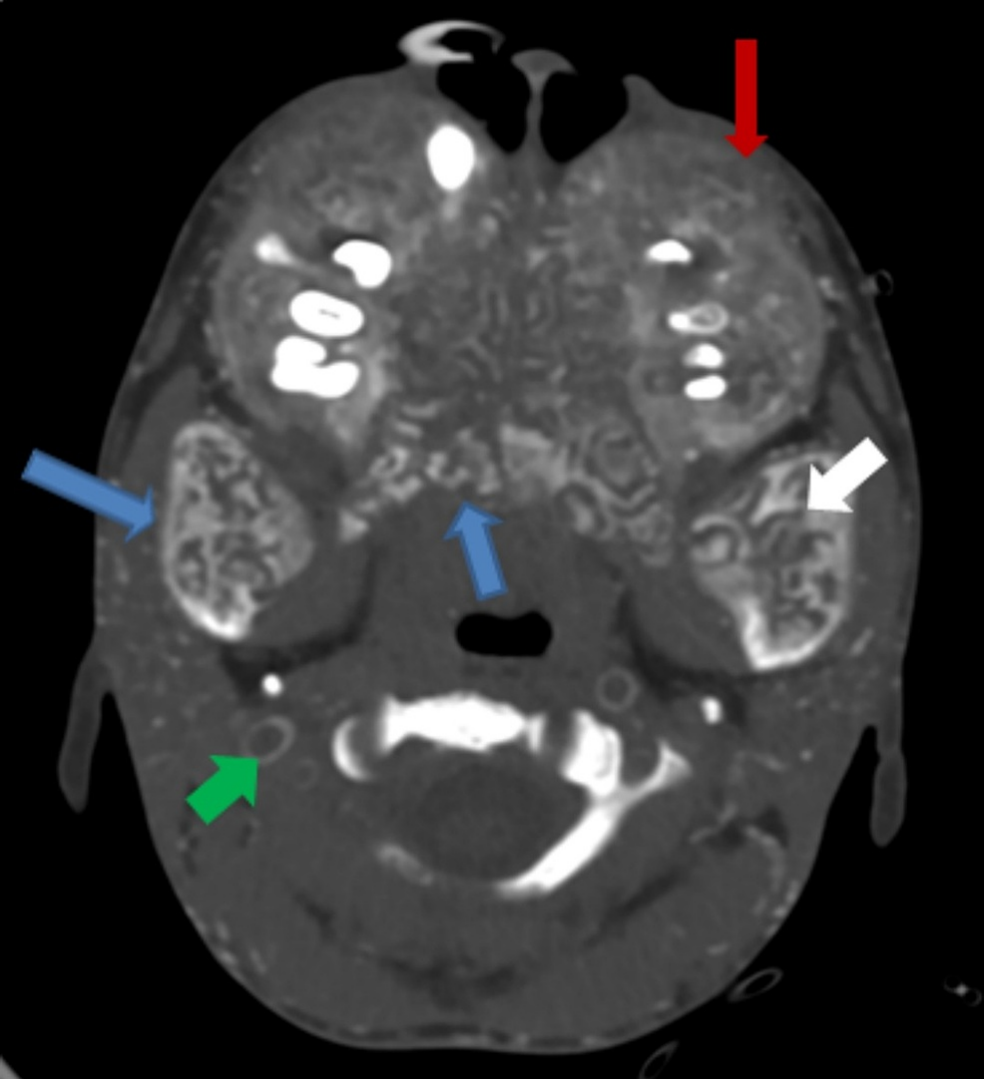
Axial bone window computed tomography The image shows the diffuse osseous expansion of the facial bones and mandible (blue arrow) with internal serpiginous lucent areas and dense trabeculae (white arrow). There is also loss of corticomedullary differentiation (red arrow), splaying of the teeth, and vascular calcification (green arrow).

**Figure 2:**
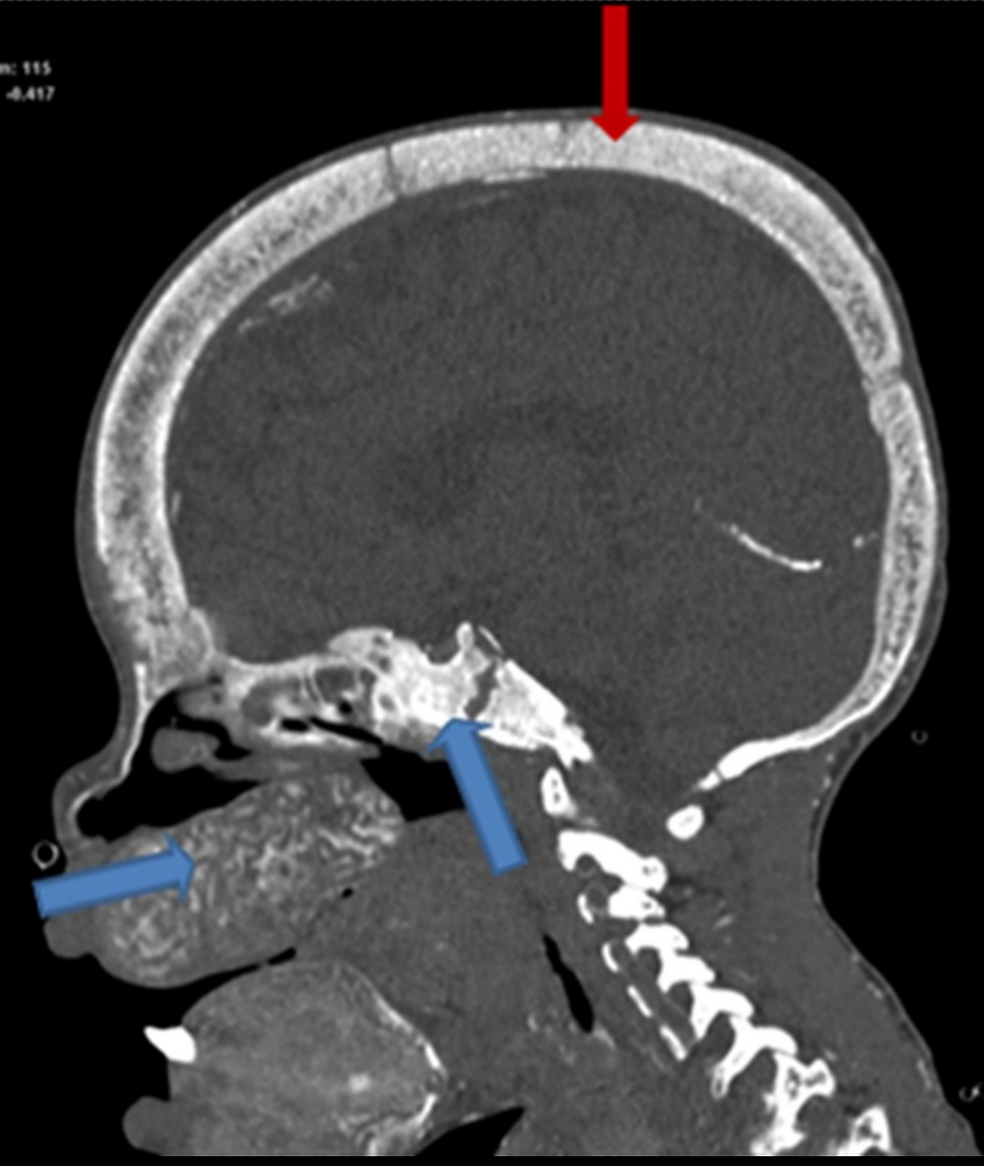
Sagittal reformatted computed tomography images bone window The image shows the diffuse expansion of the calvarial interdiploic space with multiple tiny osseous lucencies and ill definition of the inner table of the skull, creating a salt-and-pepper appearance (red arrow). The blue arrows indicate osseous expansion of the clivus and skull base.

**Figure 3:**
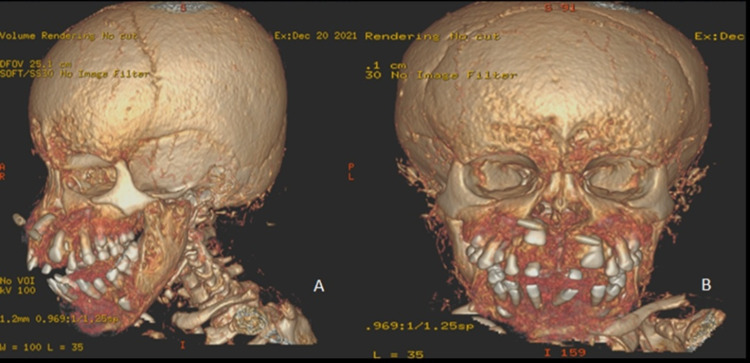
Reformatted three-dimensional computed tomography images Images show significant hypertrophy of the calvarium, maxillary, and mandibular bones with flattening of the nasal bridge. There is also splaying of the teeth.

**Figure 4:**
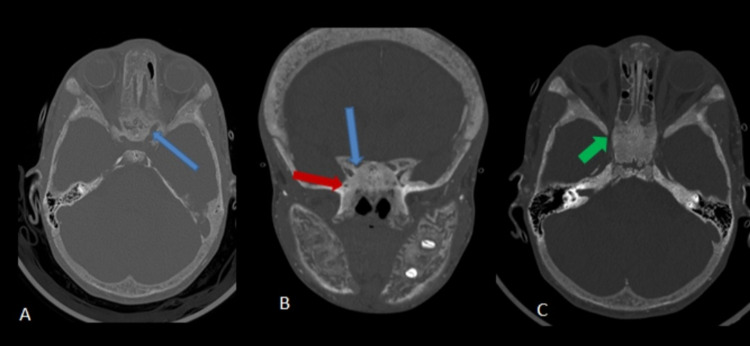
Selected CT bone window image of the skull base axial (A) and coronal (B) bone window CT images The image shows narrowing of the bilateral optic canals (blue arrow) and foramen rotundum (red arrow). There is a narrowing of the superior orbital fissure bilaterally (green arrow).

**Figure 5:**
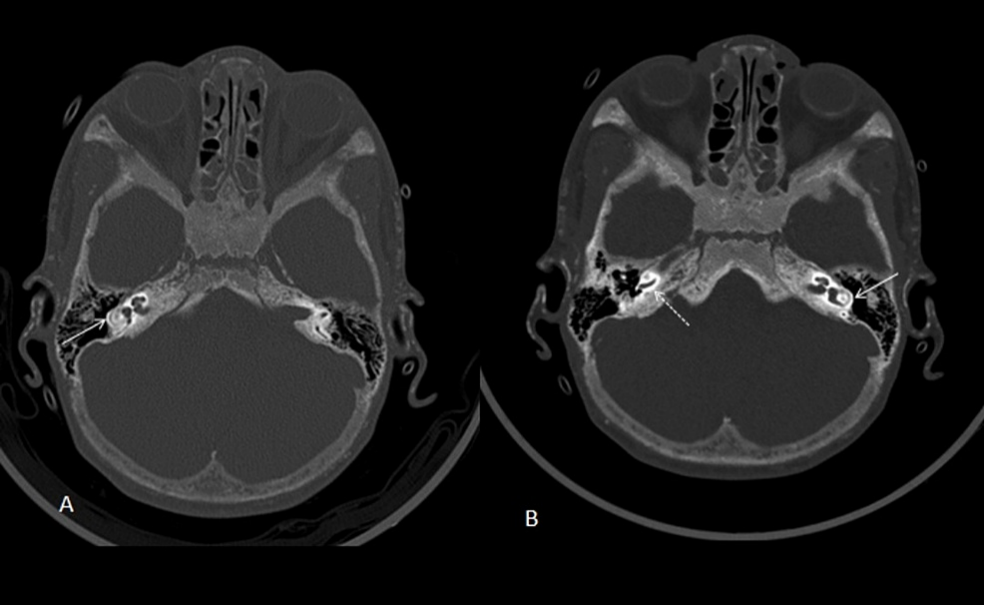
Computed tomography image bone windows shows increased density within the bony labyrinth The selected image shows increased density within the bony labyrinth (dashed arrow in B) and calcification, especially within the lateral semicircular canals (continuous arrows in A and B), representing labyrinthitis ossificans.

**Figure 6:**
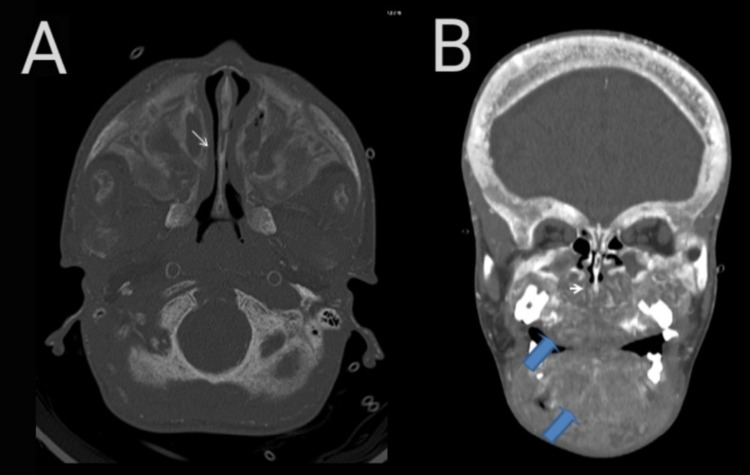
Axial (A) and coronal (B) CT images in bone window (A) shows narrowing within the mid nasal cavity (small white arrow). In (B), there is diffuse osseous expansion of the facial bones and the mandible (blue arrows).

Computed tomography (CT) of the head and neck was performed, which showed diffuse osseous and soft tissue related to hyperparathyroidism as follows: severe diffuse osseous expansion of the facial bones and mandible with internal serpiginous lucency and ground glass appearance; mild narrowing of the bilateral superior orbital fissures, mid-segment of the nasal cavity, and bilateral foramen rotundum; and high-density deposition within the membranous labyrinth, predominantly affecting the lateral semicircular canal representing labyrinthitis ossificans (Figures 1-6).

Tc-99m MIBI dual-phase parathyroid scan was also performed, but it showed no scintigraphic evidence of parathyroid adenoma.

The patient was on regular hemodialysis. After the patient was diagnosed with tertiary hyperparathyroidism, parathyroidectomy was performed smoothly without complications; the biopsy showed benign hyperplasia. She was receiving oral calcium, vitamin D supplements, and phosphate-binding medication. As for oral dysphagia, the swallowing team was consulted and educated the patient and her parents on the proper feeding positioning and techniques such as ensuring a semi-upright position with the head in the midline and a small bolus size. The maxillofacial determined that the gingival hyperplasia had improved postoperatively.

## Discussion

ULO is a rare condition that occurs as a complication of hyperparathyroidism in patients with ESKD, particularly in patients with poor adherence to hemodialysis [6]. ULO can be clinically identified by its characteristic lion-like jaw enlargement, widening of the nares, flattening of the nasal bridge, and increased interdental spacing [7]. The bony expansion that occurs in ULO occurs as a result of excess PTH in these patients, leading to unbalanced and increased activity of osteoclasts and osteoblasts [8].

ULO can have serious complications, including exophthalmos and visual loss, and, in severe cases, can even lead to life-threatening upper airway obstruction, making recognition of its features important for clinicians [1]. The first clinical feature to appear might not necessarily be the classic and extremely apparent change in facial features, as was the case with our patient. In contrast, it can be a mere complaint of facial swelling, which might be accompanied only by jaw pain [6].

Although ULO has been very rare for decades likely owing to considerable advancements in both therapeutics and diagnostics for its underlying cause, its potentially serious complications and disfigurements require urgent recognition by clinicians. This can hopefully lead to increased prevention of the disease, particularly in underserved patient groups and patients in which the classic signs are difficult to identify.

A medical history of ESRD, physical examination findings, and characteristic CT/NM imaging results are likely sufficient for the diagnosis, although some centers may proceed with bone biopsies for further confirmation.

As this disease occurs as a complication secondary to hyperparathyroidism in patients with ESRD, it is extremely rare in children. This makes our case very unique, and we did not find any radiological case report published in the literature of a patient as young as ours. Furthermore, our case shows that ULO can occur even in the pediatric age group.

CT of the head and neck showed diffuse osseous and soft tissue related to hyperparathyroidism, as well as severe diffuse osseous expansion of the facial bones and mandible with mild narrowing of the bilateral superior orbital fissures and bilateral foramen rotundum and internal serpiginous lucency and ground glass densities, all of which are expected to be observed in patients with ULO. There was also ossification of the temporal bone, which has not been observed before in this age group.

Bone resorption is radiologically manifested with a “salt-and-pepper” appearance formed by the combination of a background of sclerotic ground-glass appearance along with multiple lucencies, particularly with the diffuse loss of corticomedullary differentiation, and is the typical radiological presentation of hyperparathyroidism. A ground-glass pattern can also occur in fibrous dysplasia; however, in ULO, it is diffused and corticomedullary differentiation is typically lost [9]. Symmetrical overgrowth is another feature that suggests ULO over fibrous dysplasia and Paget's disease, which are the main differential diagnoses for ULO [7].

A nuclear parathyroid scan showed no scintigraphic evidence of parathyroid adenoma, yet the diagnosis of hyperparathyroidism was evident in laboratory studies.

Our patient did not have any clinical manifestations of cranial nerve palsy yet was suffering from dysphagia and recurrent choking episodes.

The management of ULO involves strict treatment of ESRD to prevent further metabolic derangement and management of hyperparathyroidism [6]. Dialysis is the primary treatment for ESRD. Options for hyperparathyroidism include parathyroidectomy, calcitriol-phosphate binding agents, and reduced dietary phosphate intake [10]. It is important to point out that early pharmacological control of PTH levels is an important preventive measure in the early phase of renal insufficiency [10]. The majority of patients are resistant to medical therapy alone [11]; however, performing parathyroidectomy and auto-transplantation has reportedly yielded some improvement or at least stabilization of the patient [12-13].

Our patient was receiving hemodialysis, parathyroidectomy, and pharmacological therapy to control the PTH level. The patient’s gingival hyperplasia was determined to have improved by the maxillofacial team and is expected to improve further.

## Conclusions

ULO is a rare condition with devastating complications. Therefore, awareness of it is important, particularly in patients with ESKD. The initial complaint might be facial swelling, possibly accompanied by jaw pain alone. Early prevention through adherence to dialysis and control of PTH levels is important. History, physical examination, and CT scans are very important. Imaging of the skull base and inner ear is essential for the prevention of hearing loss. Combining the medical and surgical management of underlying causes has been shown to be effective in halting progression.
